# Supplementary material for: Coiled-coil heterodimers with increased stability for cellular regulation and sensing SARS-CoV-2 spike protein-mediated cell fusion
Source: Sci Rep. 2021 Apr 28;11:9136. doi: 10.1038/s41598-021-88315-3 (PMC8080620; doi:10.1038/s41598-021-88315-3)
Supplement: Supplementary file 1 — Supplementary Information. [file 41598_2021_88315_MOESM1_ESM.pdf]

Coiled-coil heterodimers with increased stability for cellular regulation and sensing SARS-CoV-2 spike protein-mediated cell fusion

Short: **Highly stable heterodimeric parallel coiled coils**

Tjaša Plaper[1,3], Jana Aupič[1], Petra Dekleva[1], Fabio Lapenta[1,2], Mateja Manček Keber[1], Roman Jerala[1,2,*], Mojca Benčina[1,2,*]

Corresponding authors. Roman Jerala, roman.jerala@ki.si, +38614760335; Mojca Benčina, mojca.bencina@ki.si, +38614760334; (both) National Institute of Chemistry, Hajdrihova 19, SI-1001 Ljubljana, Slovenia, Fax: +38614760300.

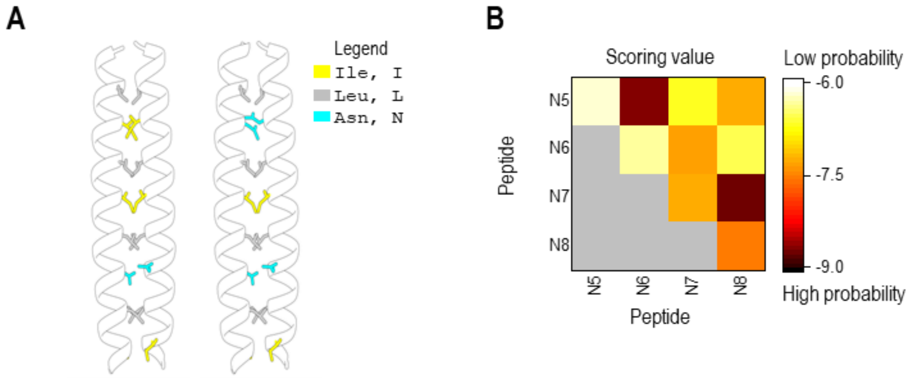

Figure S1. (**A**) Structural models of N7:N8 and P7A:P8A peptides built by the ISAMBARD modeling package. Selected amino acid side chains are shown for clarity. Polar amino acid residue Asn (cyan) is present at the *a* position of the second heptad of the N7, N8 (left). In P7A and P8A (right), additional Asn is placed at the *a* position of the fourth heptad. (**B**) Predicted orthogonality and interactions between all peptide pairs using a scoring algorithm[31]. The pairs, N5:N6 and N7:N8, were predicted to form CCs with higher stability.

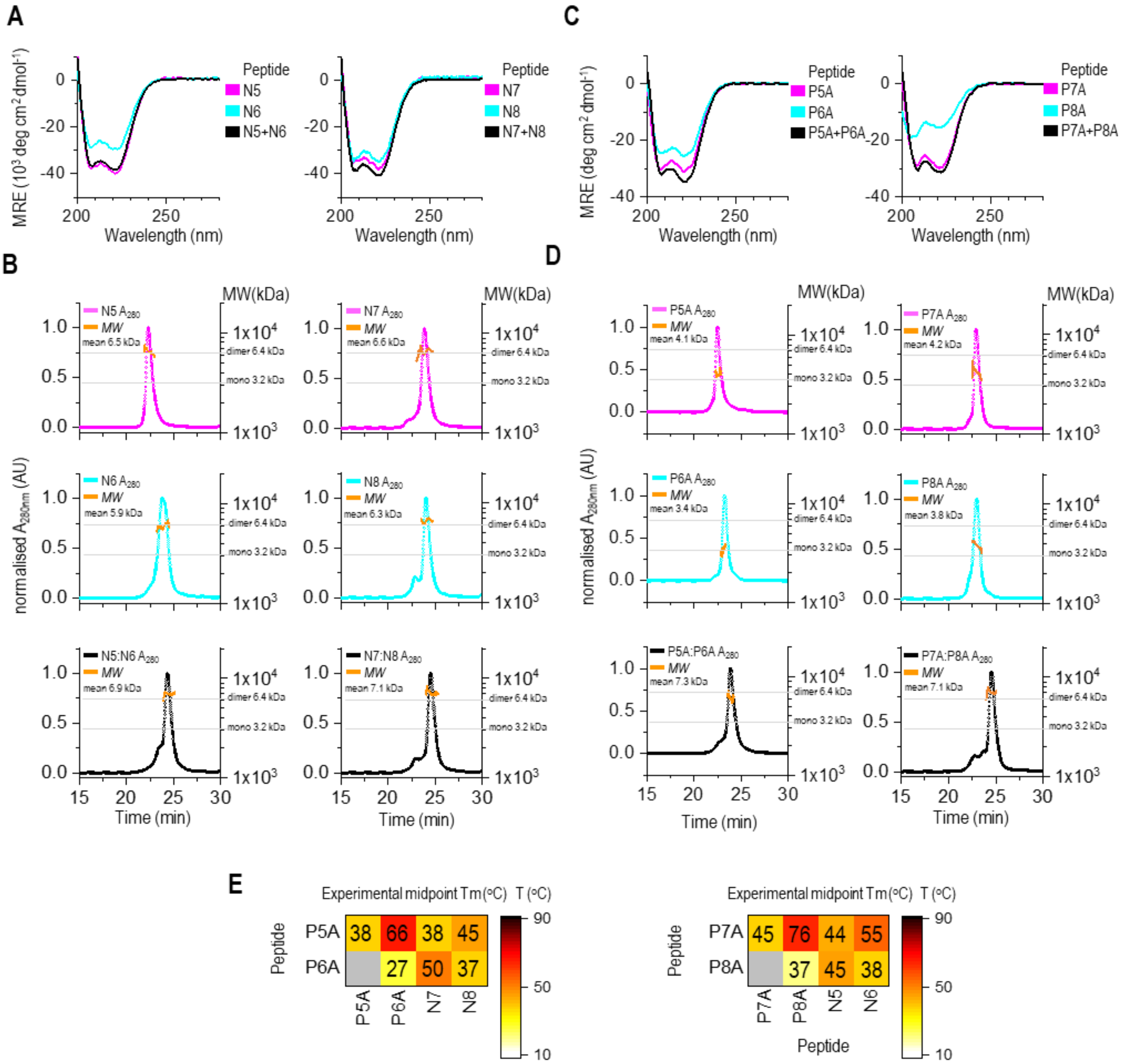

Figure S2. (**A**,**C**) Circular dichroism (CD) spectra of a 1:1 mixture of CC, N5:N6 and N7:N8 pairs, and P5A:P6A and P7A:P8A pairs (20 µM each, black). Single peptides (40 µM) are shown in cyan and magenta. The peptides and peptide mixtures resemble the characteristic α-helical spectrum at 20 ºC. The designated peptide partners exhibited a higher helical content than the peptides alone, indicating peptides' intrinsic preference for binding to their designated partners. All data were measured in Tris buffer. (**B**,**D**) Multimerization state of individual peptides and peptide pairs, N5:N6 and N7:N8, as determined by SEC-MALS. Size-exclusion chromatograms (normalized by setting the major peak maximum to 1) are presented with an overlay of the molecular weights (orange line) calculated from static light measurements. The dashed horizontal lines correspond to the expected molecular weights of a monomer and dimer. The SEC-MALS profiles of individual peptides confirm that the peptides P7A, P8A, P5A, and P6A are monomers in solution. (**E**) Heat map of the matrix of the calculated midpoint Tm from thermal denaturation scans of indicated peptide combinations.

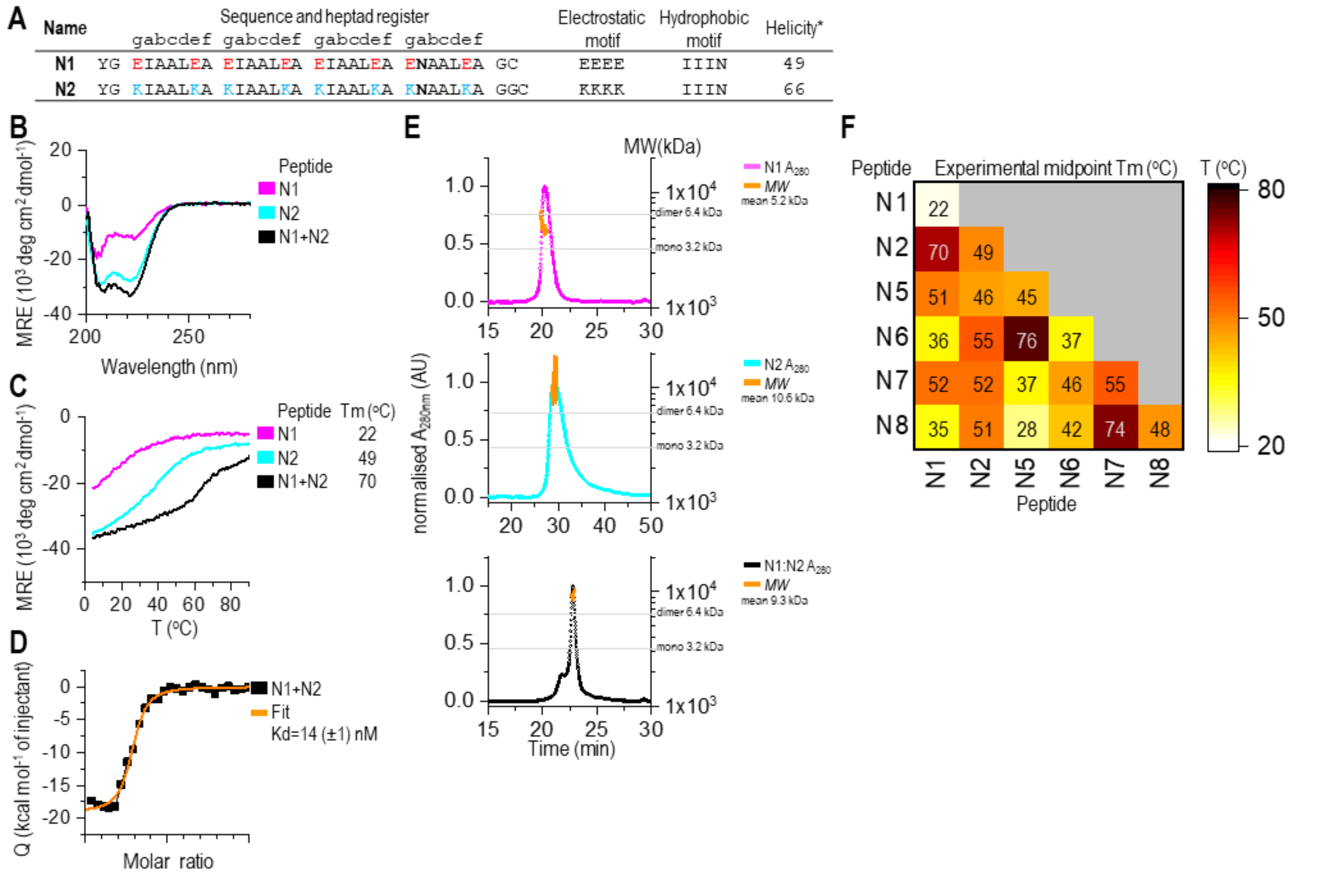

Figure S3. (**A**) Sequence of N1 and N2 peptides. (**B**) Circular dichroism (CD) spectra of a 1:1 mixture of CC, N1:N2 pairs (20 µM each, black). Single peptides (40 µM) are shown in cyan and magenta. The peptides and peptide mixtures resemble the characteristic α-helical spectrum at 20 ºC. All data were measured in Tris buffer. (**C**) Thermal denaturation profiles of peptides (40 µM; magenta and cyan) and CCs (20 µM each peptide; black) monitored by a CD signal at 222 nm. The midpoint Tm was calculated based on thermodynamic model fit [34]. (**D**) Isothermal titration calorimetry (ITC) analysis of the binding affinity of designated CC peptide pairs. The binding isotherms of heat release per injection are depicted as a function of the increasing peptide-to-peptide molar ratio. The dissociation constant, K_d^{ITC}, was calculated using the two-state dimer association model. (**E**) Multimerization state of individual peptides and peptide pairs, N1:N2, as determined by SEC-MALS. Size-exclusion chromatograms (normalized by setting the major peak maximum to 1) are presented with an overlay of the molecular weights (orange line) calculated from static light measurements. The dashed horizontal lines correspond to the expected molecular weights of a monomer and dimer. The SEC-MALS profiles of individual peptides confirm that the peptides are multimer in solution. (**F**) Heat map of the matrix of the calculated midpoint Tm from thermal denaturation scans of all peptide combinations.

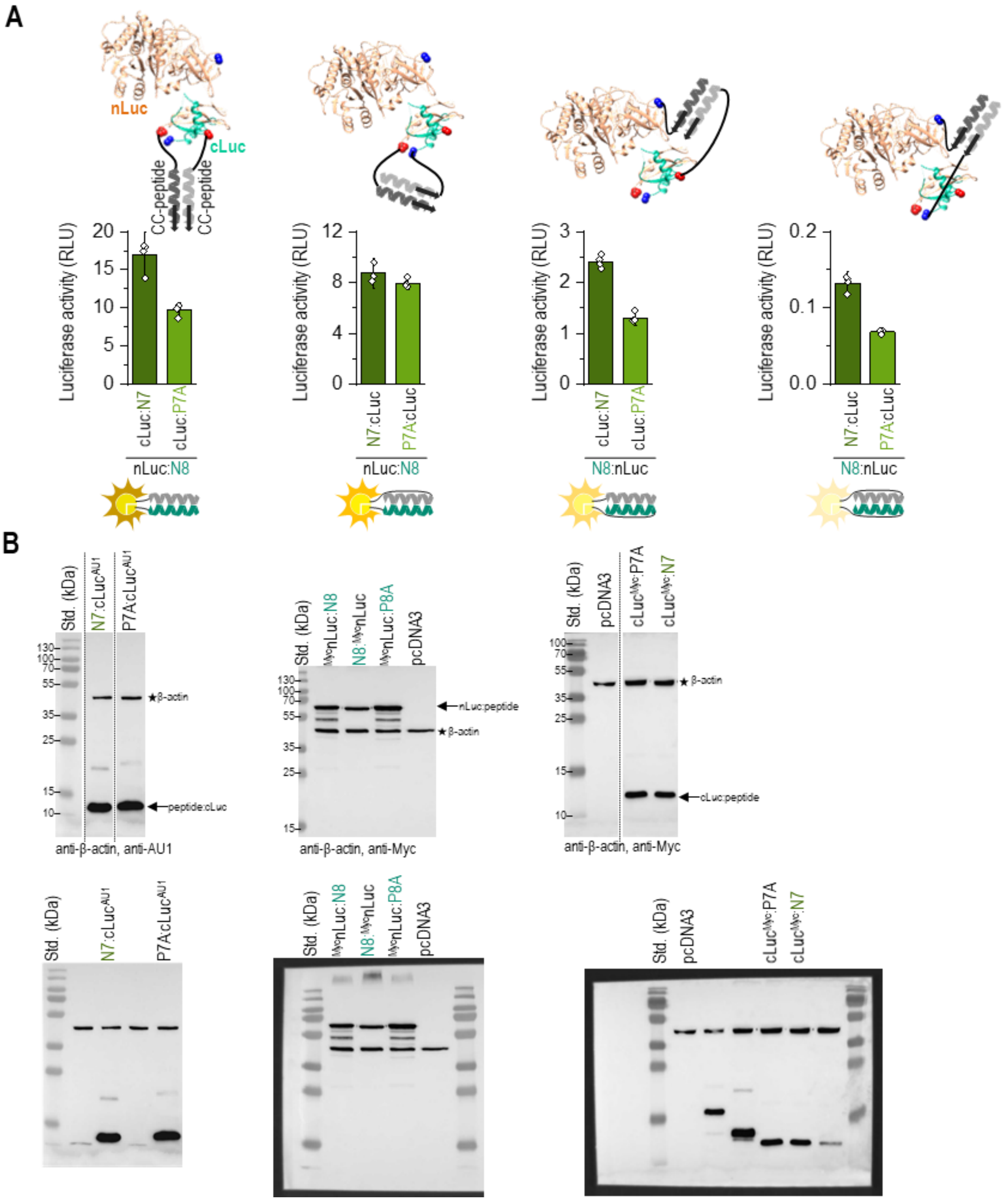

Figure S4. (**A**) Reconstitution of split luciferase in HEK293T cells. (Above) Schematic presentation of a structural model of split luciferase (brown nLuc and cyan cLuc) tethered to the N- or C-termini of the CC-forming peptide. *Note*: Linkers between CC peptides and N or C terminal of split luciferase have the same length. (Below) Luciferase activity of reconstituted CC-split luciferase 48 h after transfection of HEK293T cells with a plasmid expressing a combination of nLuc tethered to N8 (60 ng) and cLuc tethered to N7, or P7A peptides (30 ng). The values represent the means (± s.d.) from four independent cell cultures and are representative of two independent experiments. Amounts of used plasmids are indicated in **Table S2**. (**B**) Expression of CC–split luciferase determined by a western blot test. Proteins separated via SDS-PAGE were blotted on a nitrocellulose membrane and stained with anti-Myc, anti-HA, and anti-β-actin antibodies as indicated. Lines indicate the groupings from different parts of the same gel (same exposure). Below: uncropped and unprocessed images of blots are shown.

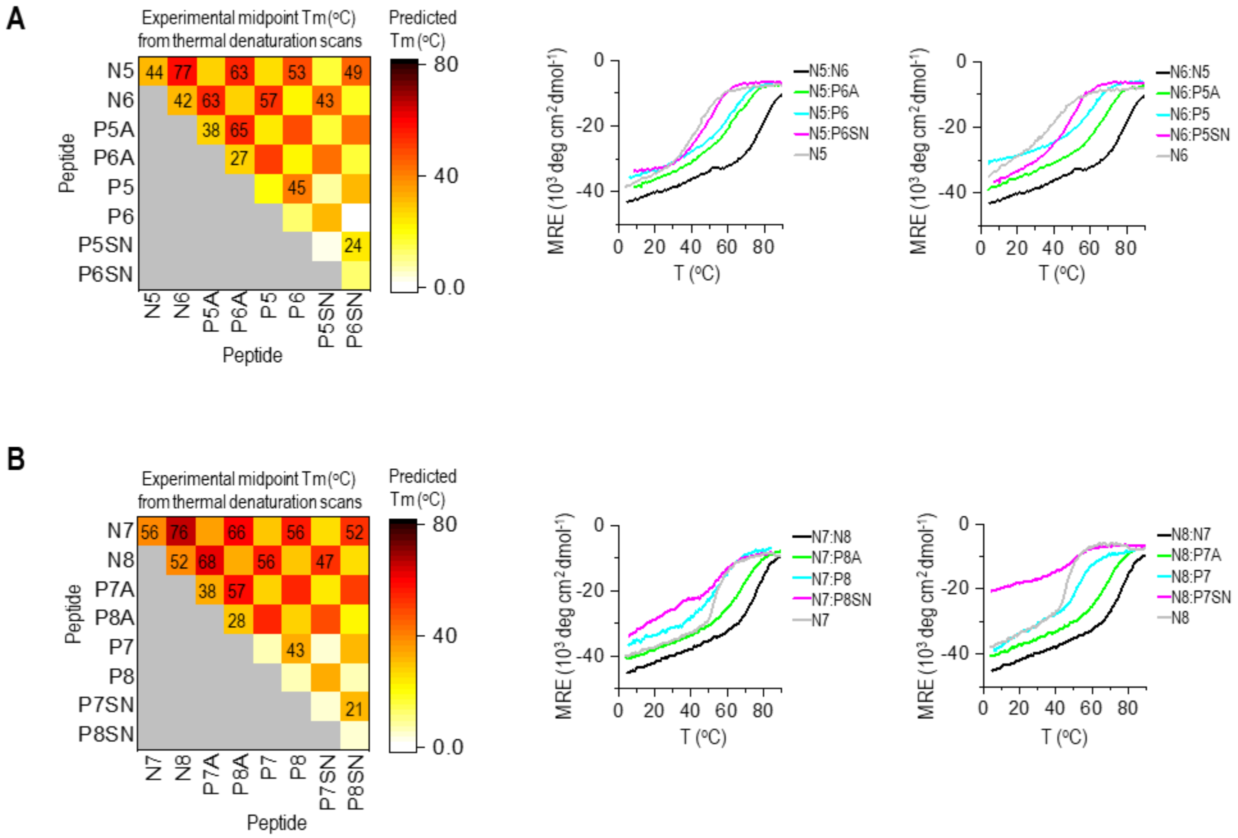

Figure S5. (**A, B**) The CC stability linear model [34] was used to predict the interactions between all possible peptide pairs. Predicted values are shown as a heat map. The experimental midpoint denaturation temperatures (Tm) of CCs and individual CC-forming peptides determined from thermal denaturation scans monitored by the CD signal at 222 nm (right) are depicted on top of the heat map of predicted Tm values.

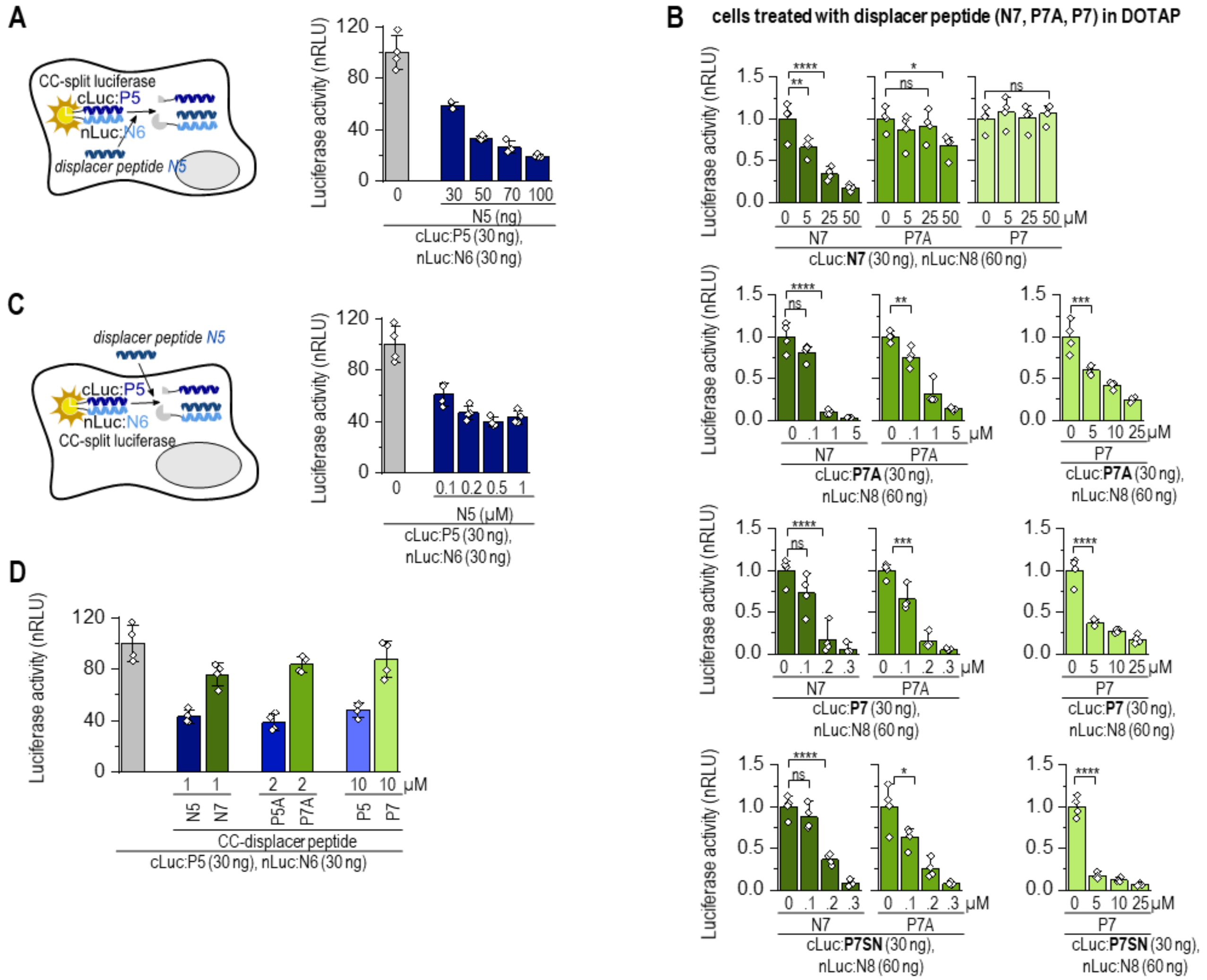

Figure S6. (A) Schematic representation of reconstituted N6:P5 split luciferase activity attenuated by N5 displacer peptide. Luciferase activity of HEK293T cells co-expressing cLuc:P5 (30 ng), nLuc:N6 (30 ng), and N5 displacer peptide (0-100 ng). **(B)** Luciferase activity of HEK293T cells transfected with plasmids expressing nLuc:N8 (60 ng) and cLuc tethered with N7, P7A, P7, or P7SN (30 ng). Forty-eight hours later, cells were treated with N7, P7A, or P7S displacer peptide (0–50 μM in DOTAP) for 2 h, and then luciferase activity was measured. **(C)** Schematic representation of reconstituted N6:P5 split luciferase activity attenuated by N5 displacer peptide, which was added to split luciferase-expressing cells. Luciferase activity of HEK293T cells (transfected with plasmids nLuc:N6 (30 ng) and cLuc tethered to P5 (30 ng)) treated with N5, P5A, P5, and P5SN peptide (0–1 μM in DOTAP) for 2 h. Cells were treated 48 h after transfection. The bars represent the means (±s.d.) from four independent cell cultures. **(D)** Luciferase activity of HEK293T cells (transfected with plasmids expressing nLuc:N6 (30 ng) and cLuc:P5 (30 ng)) treated with N7 or N5; P7A or P5A; P7 or P5 peptide (1, 2, or 10 μM in DOTAP) for 2 h. Cells were treated 48 h after transfection. The values (**A-D**) represent the means (± s.d.) from four independent cell cultures, individually transfected with the same mixture of plasmids, and are representative of two independent experiments. For amounts of plasmids, see **Table S2**. Statistical analyses and the corresponding p-values are listed in **Table S3**.

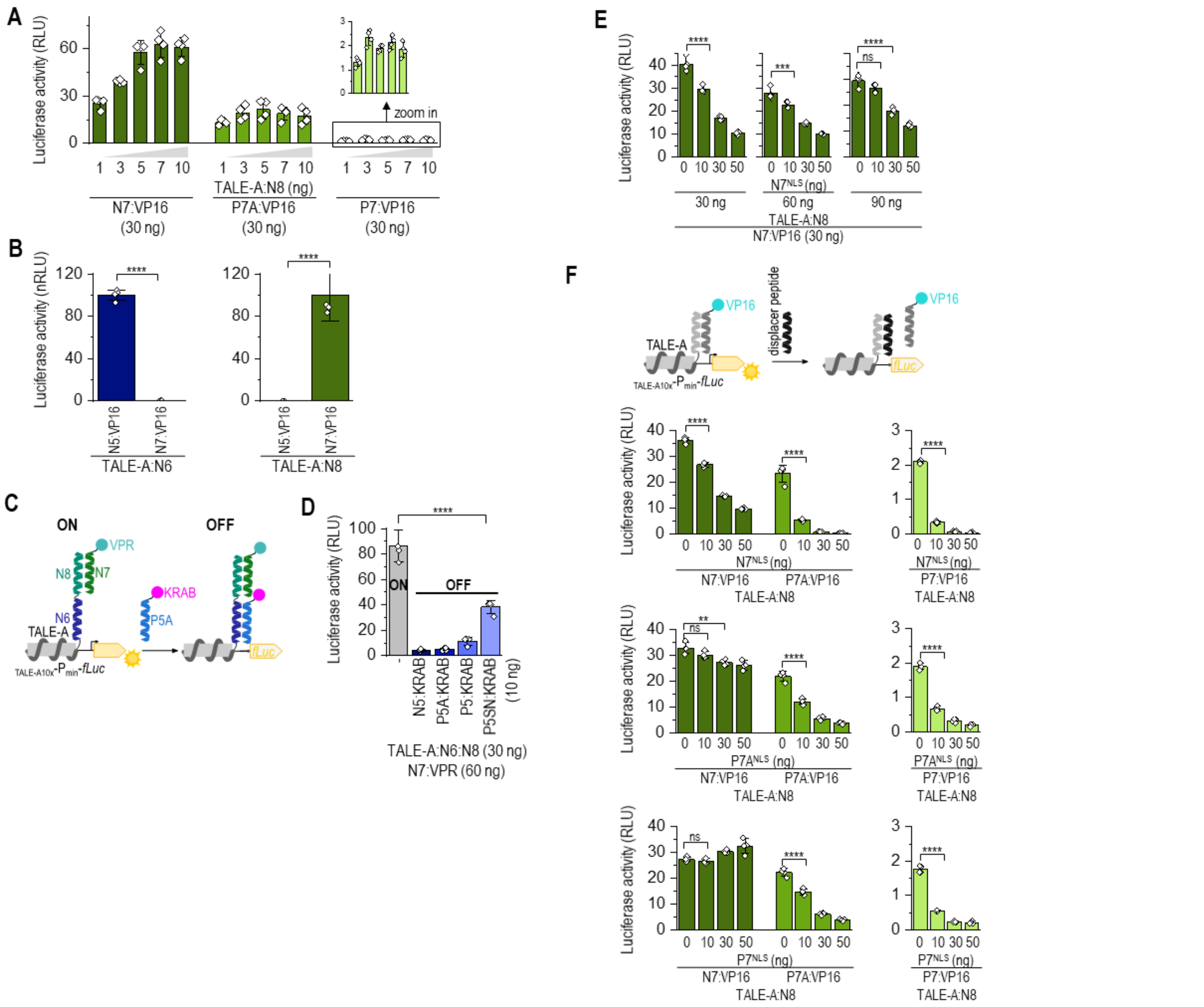

Figure S7. Reconstitution of CC–split transcription factor attenuated with complementary CC–displacer peptide. (**A**) Luciferase activity measured 48 h after transfection of HEK293T cells with plasmids expressing TALE-A:N8, CC:VP16 (30 ng) and the luciferase reporter. Zoom in depicts the reconstitution of TALE-A:N8 with P7:VP16 at more appropriate scale. **(B)** Reconstitution of CC–split transcription factor in HEK293T cells co-expressing TALE-A:N8 (30 ng); VP16 linked to N7 or N5 (30 ng) or TALE-A:N6 (30 ng) and VP16 linked to N5 or N7 (30 ng); and the luciferase reporter ($_{TALE10x}$-P$_{min}$-fLuc). Luciferase activity was measured 48 h after transfection. **(C)** Schematic representation of suppression of CC–split transcription factor activity (TALE-A:N6:N8-N7:VPR; ON state) by addition of CC:KRAB suppression domain (OFF state). **(D)** Suppression of CC–split transcription factor activity (TALE-A:N6:N8 (30 ng), N7:VPR (60 ng)) by addition of KRAB suppression domain linked to N5, P5A, P5, or P5SN (10 ng). Luciferase activity was measured 48 h after transfection. (**E**) The amount of DNA-binding domain (TALE-A:N8) has a minor impact on displacement efficacy. Luciferase activity in HEK293T cells was measured 48 h after co-transfection of plasmids expressing TALE-A:N8 (30, 60, 90 ng), N7:VP16 (30 ng), N7^{NLS} (0-50 ng) and reporter luciferase. (**F**) CC stability and CC–displacer peptide helicity determine displacement efficacy. Luciferase activity determined 48 h after transfection of HEK293T cells with plasmids expressing TALE-A:N8 (30 ng); CC:VP16 (30 ng); a CC–displacer peptide (N7^{NLS}, P7A^{NLS} or P7^{NLS}) (0-50 ng); and the reporter luciferase ($_{TALE10x}$-P$_{min}$-fLuc). The values (**A,B,D-F**) represent the means (± s.d.) from four independent cell cultures, individually transfected with the same mixture of plasmids, and are representative of two independent experiments. For amounts of plasmids, see **Table S2**. Statistical analyses and the corresponding p-values are listed in **Table S3**.

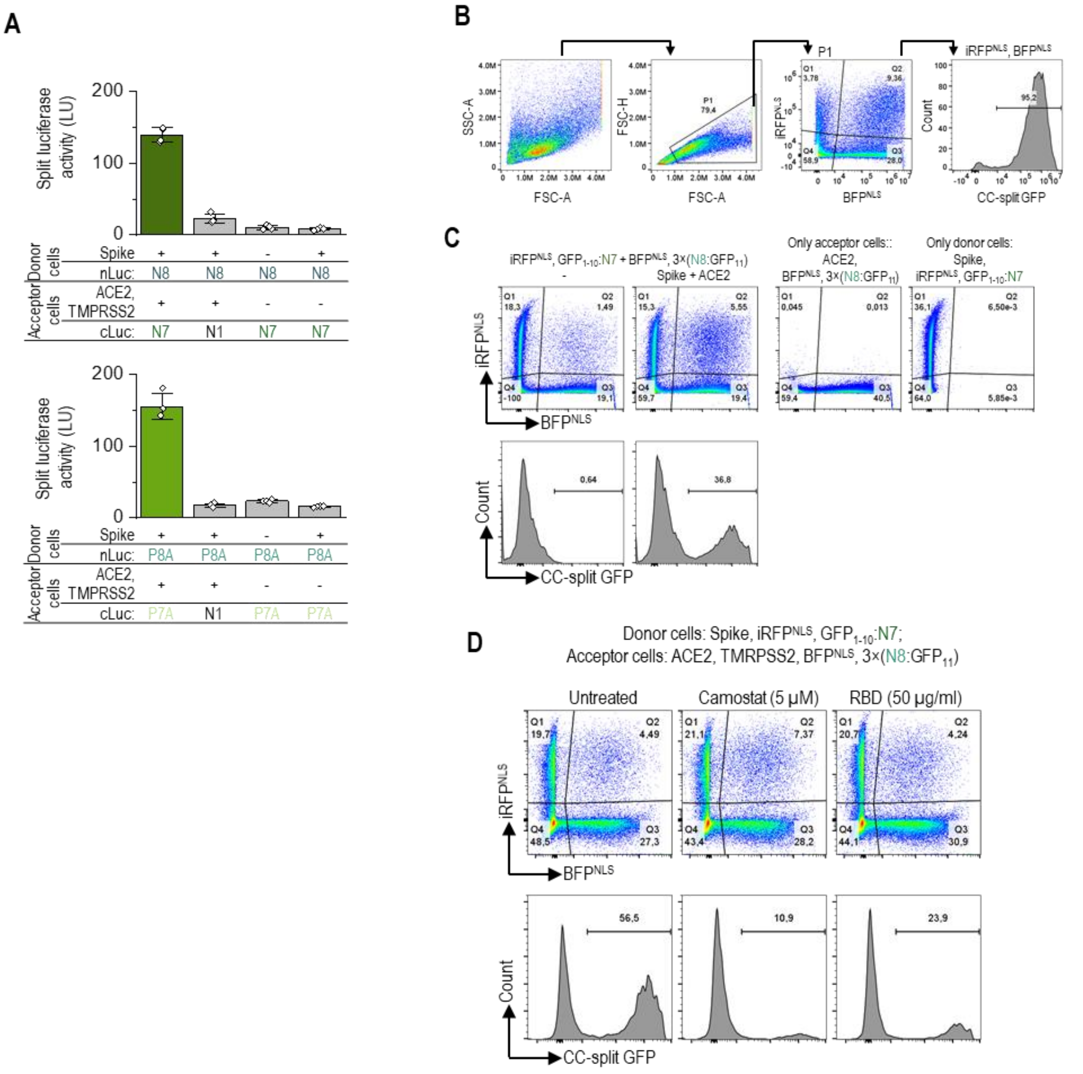

Figure S8. (**A**) Luciferase activity as an indicator of cell fusion. 24 h after transfection, donor and acceptor HEK293T cells were mixed in 1:1 ratio. Luciferase activity (A) was measured 3 h later. The values represent the means (± s.d.) from four independent cell cultures, individually transfected with the same mixture of plasmids, and are representative of two independent experiments. Donor HEK293T cells were transfected with plasmids expressing nLuc:N8 (1000 ng), with or without CoV-2 Spike-protein (10 ng) and acceptor cells expressing cLuc:N7 or cLuc:N1 as a control (1000 ng), TMPRSS2 (30 ng) with or without ACE2 (20 ng). (**B**) Flow cytometry gating strategy. The population of cells presented as pseudocolor plot (FCS-A/FCS-H) was gated for singlets and syncytia. The subset of cells, the BFP and iRFP positive, were analyzed for reconstituted CC-split GFP. The same gating strategy was used for **Fig 5E**, **Fig 6E,** and **Fig S8C, S8D**. (**C**) Flow cytometry analysis 3 h after mixing donor and acceptor cells. Formation of cell-cell fusion with split GFP reporter (GFP$_{1-10}$:N7; 3×(N8:GFP$_{11}$) was analyzed from the double BFP and iRFP positive subset of cells and presented as histogram of split GFP positive cells. Donor cells were transfected with plasmid expressing the iRFP^{NLS} (50 ng), and GFP$_{1-10}$:N7 (500 ng) with or without SARS CoV-2 Spike protein (50 ng), and the acceptor cells were expressing BFP^{NLS} (50 ng), and 3×(N8:GFP$_{11}$) (650 ng), with or without ACE2 receptor (250 ng). (**D**) Flow cytometry analysis of a mixture of donor cells expressing the SARS CoV-2 Spike protein (50 ng), iRFP^{NLS} (50 ng), and GFP$_{1-10}$:N7 (500 ng) and the acceptor cells expressing ACE2 receptor (250 ng), TMPRSS2 (50 ng), BFP^{NLS} (50 ng), and 3×(N8:GFP$_{11}$) (650 ng). Formation of cell-cell fusion with split GFP reporter (GFP$_{1-10}$:N7; 3×(N8:GFP$_{11}$) was analyzed from the populations of cells positive for iRFP and BFP. Histograms present percent of reconstituted split GFP for double iRFP and BFP positive 3 h after mixing donor and acceptor cells. Representative results of two independent experiments are shown. For amounts of plasmids, see **Table S2**.

Table S1. Sequences of orthogonal 5:6 and 7:8 N- and P-type peptides.

Name		Sequence and heptad register gabcdef gabcdef gabcdef gabcdef		Electrostatic motif	Hydrophobic motif	Helicity*
**N7**	Y	EIAALEA KNAALKA EIAALEA KIAALKA	GC	EKEK	INII	65
**P7A**	YG	EIAALEA KNAALKA EIAALEA KNAALKA	GC	EKEK	ININ	43
P7	SPED	EIQALEE KNAQLKQ EIAALEE KNQALKY	G	EKEK	ININ	13
P7SN		EIQQLEE KNSQLKQ EISQLEE KNQELKY	G	EKEK	ININ	3
**N8**	Y	KIAALKA ENAALEA KIAALKA EIAALEA	GC	KEKE	INII	59
**P8A**	YG	KIAALKA ENAALEA KIAALKA ENAALEA	GGC	KEKE	ININ	38
P8	SPED	KIAQLKE ENQQLEQ KIQALKE ENAALEY	G	KEKE	ININ	11
P8SN		KISELKE ENQQLEQ KIQQLKE ENSQLEY	G	KEKE	ININ	4
**N5**	Y	EIAALEA KIAALKA KNAALKA EIAALEA	GC	EKKE	IINI	61
**P5A**	YG	ENAALEA KIAALKA KNAALKA EIAALEA	GC	EKKE	NINI	51
P5	SPED	ENAALEE KIAQLKQ KNAALKE EIQALEY	G	EKKE	NINI	20
P5SN		ENSQLEE KISQLKQ KNSELKE EIQQLEY	G	EKKE	NINI	4
**N6**	Y	KIAALKA EIAALEA ENAALEA KIAALKA	GC	KEEK	IINI	60
**P6A**	YG	KNAALKA EIAALEA ENAALEA KIAALKA	GGC	KEEK	NINI	45
P6	SPED	KNAALKE EIQALEE ENQALEE KIAQLKY	G	KEEK	NINI	16
P6SN		KNSELKE EIQQLEE ENQQLEE KISELKY	G	KEEK	NINI	4

Legend: negative and positive amino acid residues are indicated with red and blue letters, respectively; asparagine is bolded;
* indicates that the value was calculated according to the method described by Agadir et al. [32].

Table S2. Amounts of transfected plasmids for HEK293T cells in each well of a multi-well plate. The empty pcDNA3 plasmid vector was used to equalize total DNA amounts up to 250 ng (for 96-well plate), 2500 ng (for 6-well plate), 1000 ng (for 8- and 12-well plate).

Input plasmids	Amount [ng]
**Figure 1G (96-well plate)**	
cLuc:N7, or N7:cLuc	30
nLuc:N8, or N8:nLuc	30
phRL-TK	5
**Figures 1H, 1I (96-well plate)**	
cLuc:N7, cLuc:P7A, cLuc:N5, or cLuc:P5A	10-70
nLuc:N8, or nLuc:N6	30
phRL-TK	5
**Figure 1J (96-well plate)**	
cLuc:N7, cluc:N5, cLuc:P7A, or cLuc:P5A	30
nLuc:N8, or nLuc:N6	30
phRL-TK	5
**Figures 2A, 2B (96-well plate)**	
cLuc:N7, cLuc:P7A, cLuc:P7, or cLuc:P7SNcLuc:N5, cLuc:P5A, cLuc:P5, or cLuc:P5SN	10-90
nLuc:N8, or nLuc:N6	30
phRL-TK	5
**Figures 2C, 2D (96-well plate)**	
cLuc:N7, cLuc:P7A, cLuc:P7, cLuc:P7SN, cLuc:N5, cLuc:P5A, cLuc:P5, or cLuc:P5SN	10
nLuc:N8, or nLuc:N6	30
phRL-TK	5
**Figure 3C (96-well plate)**	
cLuc:N7, cLuc:P7A, cLuc:P7, or cLuc:P7SN	30
nLuc:N8	60
N7 displacer peptide	0, 80, 155
phRL-TK	50

Input plasmids	Amount [ng]
**Figure 3E, S6B (96-well plate)**	
cLuc:N7, cLuc:P7A, cLuc:P7, or cLuc:P7SN	30
nLuc:N8	60
phRL-TK	5
**Figure 3G (96-well plate)**	
cLuc:P7	10
nLuc:N8	30
phRL-TK	5
**Figure 4B (96-well plate)**	
TALE-A:NLS:N8, or TALE-A:NLS:N6	30
N7:NLS:VP16, N5:NLS:VP6, P7A:NLS:VP16 or P5A:NLS:VP16	1-100
phRL-TK	5
**Figure 4D (96-well plate)**	
TALE-A:NLS:N6:N8	30
N7:VP16	30
N5:KRAB, P5A:KRAB, P5:KRAB, or P5SN:KRAB	0-3
phRL-TK	5
**Figure 4H (96-well plate)**	
TALE-A:NLS:N6:N8	30
N7:NLS:VP16	30
P5:KRAB	1
N5:^{NLS} displacer peptide	0-90
phRL-TK	5
**Figure 4F (96-well plate)**	
TALE-A:NLS:N6:N8	30
P7A:VP16	30
N7^{NLS} displacer peptide	0-70
phRL-TK	5

Input plasmids	Amount [ng]
**Figure 5B, 6B, 6D, S8A**	
**Acceptor cells (6-well plate)**	
cLuc:N7, or cLuc:P7A, or cLuc:N1	1000
ACE2	0, 20
TMPRSS2	0, 30
**Donor cells (6-well plate)**	
nLuc:N8, or nLuc:P8A	1000
S-protein	0, 10
**Figure 5D**	
**Acceptor cells (8-well plate)**	
N8:GFP_{11}, 3x(N8:GFP_{11}), or P8:GFP_{11}	100
ACE2	40
TMPRSS2	40
BFP^{NLS}	120
**Donor cells (8-well plate)**	
S-protein	25
GFP_{1-10}:N7, or GFP_{1-10}:P7	100
$mCherry^{NLS}$	500
**Figure 5E, 6E, S8B, S8C, S8D (12-well plate)**	
**Acceptor cells (12-well plate)**	
N8:GFP_{11}, 3x(N8:GFP_{11}), or P8:GFP_{11}	650
ACE2	250
TMPRSS2	50
BFP^{NLS}	50
**Donor cells (12-well plate)**	
S-protein	50
GFP_{1-10}:N7, or GFP_{1-10}:P7,	500
$iRFP^{NLS}$	50

Input plasmids	Amount [ng]
**Figure S4A (96-well plate)**	
cLuc:N7, cluc:P7A, N7:cLuc, or P7A:cLuc	30
nLuc:N8, or N8:nLuc	60
phRL-TK	5
**Figure S6A (96-well plate)**	
cLuc:P5	30
N5	0-100
nLuc:N6	30
phRL-TK	5
**Figure S6C, S6D (96-well plate)**	
cLuc:P5	30
nLuc:N6	30
phRL-TK	5
**Figure S7A (96-well plate)**	
TALE-A:NLS:N8	1-10
N7:NLS:VP16, P7A:NLS:VP16 or P7:NLS:VP16	30
phRL-TK	5
**Figure S7B (96-well plate)**	
TALE-A:N8 or TALE-A:N6	30
N7:VP16 or N5:VP16	30
phRL-TK	5
**Figure S7E (96-well plate)**	
TALE-A:N8	30, 60, 90
N7:VP16	30
$N7^{NLS}$	0-50
phRL-TK	5
**Figure S7F (96-well plate)**	
TALE-A:N8	30
N7:VP16 P7A:VP16 P7:VP16	30
$N7^{NLS}$ $P7A^{NLS}$ $P7^{NLS}$	0-50
phRL-TK	5
**Figure S7C (96-well plate)**	
TALE-A:N6:N8	30
N7:VPR	60
N5:KRAB P5A:KRAB P5:KRAB P5SN:KRAB	10
phRL-TK	5

Table S3. Statistical analyses data.

Figures	Test details	Significance	Summary	P-value
Fig 3C	**One-way ANOVA; Dunnett`s multiple comparisons test**			
	nLuc:N8 cLuc:N7; N7			
	0 *vs.* 80	No	ns	0.6788
	0 vs. 155	No	ns	0.2292
	nLuc:N8 cLuc:P7A; N7			
	0 vs. 80	Yes	****	< 0,0001
	0 vs. 155	Yes	****	< 0,0001
	nLuc:N8 cLuc:P7; N7			
	0 vs. 80	Yes	****	< 0,0001
	0 vs. 155	Yes	****	< 0,0001
	nLuc:N8 cLuc:P7SN; N7			
	0 vs. 80	Yes	****	< 0,0001
	0 vs. 155	Yes	****	< 0,0001
Fig 3E	**One-way ANOVA; Dunnett`s multiple comparisons test**			
	cLuc:N7 nLuc:N8; N7			
	0 vs. 0.1	No	ns	0.9861
	0 vs. 0.2	No	ns	0.9997
	0 vs. 0.4	No	ns	0.8104
	0 vs. 1	No	ns	> 0,9999
	0 vs. 2	No	ns	0.9997
	0 vs. 5	No	ns	0.5602
	0 vs. 10	Yes	**	0.0037
	**One-way ANOVA Dunnett`s multiple comparisons test**			
	cLuc:P7A nLuc:N8; N7			
	0 *vs.* n (for all tested amounts)	Yes	****	< 0,0001
	cLuc:P7 nLuc:N8; N7			
	0 *vs.* n (for all tested amounts)	Yes	****	< 0,0001
	cLuc:P7SN nLuc:N8; N7			
	0 *vs.* n (for all tested amounts)	Yes	****	< 0,0001
Fig S5B	**One-way ANOVA; Dunnett`s multiple comparisons test**			
	nLuc:N8 cLuc:N7; N7			
	0 *vs.* 5	Yes	**	0.0072
	0 *vs.* 25	Yes	****	< 0,0001
	0 *vs.* 50	Yes	****	< 0,0001
	nLuc:N8 cLuc:N7; P7A			
	0 *vs.* 5	No	ns	0.5576
	0 *vs.* 25	No	ns	0.8284
	0 *vs.* 50	Yes	*	0.0445
	nLuc:N8 cLuc:N7; P7			
	0 *vs.* 5	No	ns	0.7673
	0 *vs.* 25	No	ns	0.9968
	0 *vs.* 50	No	ns	0.8588
	nLuc:N8 cLuc:P7A; N7			
	0 *vs.* 0.1	No	ns	0.0575
	0 *vs.* 1	Yes	****	< 0,0001
	0 *vs.* 5	Yes	****	< 0,0001
	nLuc:N8 cLuc: P7A; P7A			
	0 *vs.* 0.1	Yes	**	0.009
	0 *vs.* 1	Yes	****	< 0,0001
	0 *vs.* 5	Yes	****	< 0,0001
	nLuc:N8 cLuc: P7A; P7			
	0 *vs.* 0.1	Yes	***	0.0003
	0 *vs.* 1	Yes	****	< 0,0001
	0 *vs.* 5	Yes	****	< 0,0001
	nLuc:N8 cLuc:P7; N7			
	0 *vs.* 0.1	No	ns	0.1021
	0 *vs.* 0.2	Yes	****	< 0,0001
	0 *vs.* 0.3	Yes	****	< 0,0001
	nLuc:N8 cLuc: P7; P7A			
	0 *vs.* 0.1	Yes	***	0.0006
	0 *vs.* 0.2	Yes	****	< 0,0001
	0 *vs.* 0.3	Yes	****	< 0,0001
	nLuc:N8 cLuc: P7; P7			
	0 *vs.* n (for all tested amounts)	Yes	****	< 0,0001
	nLuc:N8 cLuc:P7SN; N7			
	0 *vs.* 0.1	No	ns	0.276
	0 *vs.* 0.2	Yes	****	< 0,0001
	0 *vs.* 0.3	Yes	****	< 0,0001
	nLuc:N8 cLuc: P7SN; P7A			
	0 *vs.* 0.1	Yes	*	0.0167
	0 *vs.* 0.2	Yes	****	< 0,0001
	0 *vs.* 0.3	Yes	****	< 0,0001
	nLuc:N8 cLuc: P7SN; P7			
	0 *vs.* n (for all tested amounts)	Yes	****	< 0,0001

Figures	Test details	Significance	Summary	P-value
Fig 4D	**One-way ANOVA; Dunnett`s multiple comparisons test**			
	TALE-A:N6:N8 N7:VP16, N5:KRAB			
	0 *vs.* n (for all tested amounts)	Yes	****	< 0,0001
	TALE-A:N6:N8 N7:VP16, P5A:KRAB			
	0 *vs.* n (for all tested amounts)	Yes	****	< 0,0001
	TALE-A:N6:N8 N7:VP16, P5:KRAB			
	0 *vs.* n (for all tested amounts)	Yes	**	0.001
	TALE-A:N6:N8 N7:VP16, P5SN:KRAB			
	0 *vs.* n (for all tested amounts)	Yes	****	< 0,0001
Fig 4F	**One-way ANOVA; Dunnett`s multiple comparisons test**			
	TALE-A:N6:N8 P7A:VP16, $N7^{NLS}$:KRAB			
	0 *vs.* n (for all tested amounts)	Yes	****	< 0,0001
Fig 4H	**One-way ANOVA; Dunnett`s multiple comparisons test**			
	- *vs.* 0	Yes	****	< 0,0001
	- *vs.* 10	Yes	****	< 0,0001
	- *vs.* 30	No	ns	0.2404
	- *vs.* 50	No	ns	0.2701
	- *vs.* 70	Yes	***	0.0004
Fig S6B	**Paired t-test, two-tailed**			
	TALE-A:N6 N5:VP16 vs. N7:VP16	Yes	****	< 0,0001
	TALE-A:N6 N8:VP16 vs. N7:VP16	Yes	****	< 0,0001
Fig S6C	**One-way ANOVA; Dunnett`s multiple comparisons test**			
	TALE-A:N8 N7:VP16, $N7^{NLS}$			
	0 *vs.* n (for all tested amounts)	Yes	****	< 0,0001
	TALE-A:N8 N7:VP16, $N7^{NLS}$			
	0 *vs.* n (for all tested amounts)	Yes	****	< 0,0001
	TALE-A:N8 N7:VP16, $N7^{NLS}$			
	0 *vs.* 10	No	ns	0.0638
	0 *vs.* 30	Yes	****	< 0,0001
	0 *vs.* 50	Yes	****	< 0,0001
Fig S6D	**One-way ANOVA; Dunnett`s multiple comparisons test**			
	TALE-A:N8 N7:VP16, $N7^{NLS}$			
	0 *vs.* n (for all tested amounts)	Yes	****	< 0,0001
	TALE-A:N8 P7A:VP16, $N7^{NLS}$			
	0 *vs.* n (for all tested amounts)	Yes	****	< 0,0001
	TALE-A:N8 P7:VP16, $N7^{NLS}$			
	0 *vs.* n (for all tested amounts)	Yes	****	< 0,0001
	TALE-A:N8 N7:VP16, $P7A^{NLS}$			
	0 *vs.* 10	No	ns	0.086
	0 *vs.* 30	Yes	**	0.0011
	0 *vs.* 50	Yes	***	0.0002
	TALE-A:N8 P7A:VP16, $P7A^{NLS}$			
	0 *vs.* n (for all tested amounts)	Yes	****	< 0,0001
	TALE-A:N8 P7:VP16, $P7A^{NLS}$			
	0 *vs.* n (for all tested amounts)	Yes	****	< 0,0001
	TALE-A:N8 N7:VP16, $P7^{NLS}$			
	0 *vs.* 10	No	ns	0.9364
	0 *vs.* 30	No	ns	0.0507
	0 *vs.* 50	Yes	**	0.0018
	TALE-A:N8 P7A:VP16, $P7^{NLS}$			
	0 *vs.* n (for all tested amounts)	Yes	****	< 0,0001
	TALE-A:N8 P7:VP16, $P7^{NLS}$			
	0 *vs.* n (for all tested amounts)	Yes	****	< 0,0001
Fig S6F	**One-way ANOVA; Dunnett`s multiple comparisons test**			
	TALE-A:N8 N7:VPR			
	- *vs.* N5:KRAB	Yes	****	< 0,0001
	- *vs.* P5A:KRAB	Yes	****	< 0,0001
	- *vs.* P5:KRAB	Yes	****	< 0,0001
	- *vs.* P5SN:KRAB	Yes	****	< 0,0001
Fig 5B	**Paired t-test, two-tailed**			
	nLuc:N8 cLuc:N7 vs. nLuc:P8A cLuc:P7A	No	ns	0.8335
Fig 6B	**One-way ANOVA; Dunnett`s multiple comparisons test**			
	Spike nLuc:P8A, TMRSS2 ACE2 cLuc:P7A, Camostat			
	0 *vs.* n (for all tested amounts)	Yes	****	< 0,0001
Fig 6D	**One-way ANOVA; Dunnett`s multiple comparisons test**			
	Spike nLuc:P8A, TMRSS2 ACE2 cLuc:P7A, RBD			
	0 *vs.* n (for all tested amounts)	Yes	****	< 0,0001

Table S4. The list of plasmids used in this study.

Plasmid name (amino acid sequence)
Myc:N7
M EQKLISEEDL GEIAALEAKNAALKAEIAALEAKIAALKAGY*
red: Myc tag; black: N7
Myc:P7A
M EQKLISEEDL GEIAALEAKNAALKAEIAALEAKNAALKAGC*
red: Myc tag; black: P7
Myc:P7
M EQKLISEEDL EIQALEEKNAQLKQEIAALEEKNQALKYG*
red: Myc tag; black: P7
Myc:P7SN
M EQKLISEEDL EIQQLEEKNSQLKQEISQLEEKNQELKYG*
red: Myc tag; black: P7SN
Myc:N7Q
M EQKLISEEDL GEIAALEQKNAALKQEIAALEQKIAALKQGC*
red: Myc tag; black: N7Q
N7:gs linker:cLuc:gs linker:HA
M GEIAALEAKNAALKAEIAALEAKIAALKAGY GGSGGGGSGGS TMTEKEIVDYVASQVTTAKKLRGGVVFVDEVPKGLTGKLDARKIREILIKAKKGGKIAVNS GSG<br>YPYDVPDYA*
black: N7; dark blue: gs linker; yellow: C terminal of split Luciferase cLuc; green:HA tag
N7Q:gs linker:cLuc:gs linker: HA
M GEIAALEQKNAALKQEIAALEQKIAALKQGC GGSGGGGSGGS TMTEKEIVDYVASQVTTAKKLRGGVVFVDEVPKGLTGKLDARKIREILIKAKKGGKIAVNS GSG<br>YPYDVPDYA*
black: N7Q; dark blue: gs linker; yellow: C terminal of split Luciferase cLuc; green:HA tag
P7A:gs linker:cLuc:gs linker:HA
M GEIAALEAKNAALKAEIAALEAKNAALKAGC GGSGGGGSGGS TMTEKEIVDYVASQVTTAKKLRGGVVFVDEVPKGLTGKLDARKIREILIKAKKGGKIAVN SGSG<br>YPYDVPDYA*
black: P7A; dark blue: gs linker; yellow: C terminal of split Luciferase cLuc; green:HA tag
P7:gs linker:cLuc:gs linker:HA
M EIQALEEKNAQLKQEIAALEEKNQALKYG GGSGGGGSGGS TMTEKEIVDYVASQVTTAKKLRGGVVFVDEVPKGLTGKLDARKIREILIKAKKGGKIAVN SGSG<br>YPYDVPDYA*
black: P7; dark blue: gs linker; yellow: C terminal of split Luciferase cLuc; green:HA tag
P7SN:gs linker:cLuc:gs linker:HA
M EIQQLEEKNSQLKQEISQLEEKNQELKYG GGSGGGGSGGS TMTEKEIVDYVASQVTTAKKLRGGVVFVDEVPKGLTGKLDARKIREILIKAKKGGKIAVN SGSG<br>YPYDVPDYA*
black: P7SN; dark blue: gs linker; yellow: C terminal of split Luciferase cLuc; green:HA tag
cLuc:Myc:N7
M TMTEKEIVDYVASQVTTAKKLRGGVVFVDEVPKGLTGKLDARKIREILIKAKKGGKIAVNS EQKLISEEDL GEIAALEAKNAALKAEIAALEAKIAALKAGY
yellow: C terminal of split Luciferase cLuc; red:Myc tag; dark blue: gs linker; black: N7
cLuc:Myc: N7Q
M TMTEKEIVDYVASQVTTAKKLRGGVVFVDEVPKGLTGKLDARKIREILIKAKKGGKIAVNS EQKLISEEDL GEIAALEQKNAALKQEIAALEQKIAALKQGC
yellow: C terminal of split Luciferase cLuc; red:Myc tag; dark blue: gs linker; black: N7
cLuc:Myc: P7A
M TMTEKEIVDYVASQVTTAKKLRGGVVFVDEVPKGLTGKLDARKIREILIKAKKGGKIAVN EQKLISEEDL GEIAALEAKNAALKAEIAALEAKNAALKAGC
yellow: C terminal of split Luciferase cLuc; red:Myc tag; dark blue: gs linker; black: N7
cLuc:Myc: P7
M TMTEKEIVDYVASQVTTAKKLRGGVVFVDEVPKGLTGKLDARKIREILIKAKKGGKIAVN EQKLISEEDL EIQALEEKNAQLKQEIAALEEKNQALKYG
yellow: C terminal of split Luciferase cLuc; red:Myc tag; dark blue: gs linker; black: N7
cLuc:Myc: P7SN
M TMTEKEIVDYVASQVTTAKKLRGGVVFVDEVPKGLTGKLDARKIREILIKAKKGGKIAVN EQKLISEEDL EIQQLEEKNSQLKQEISQLEEKNQELKYG
yellow: C terminal of split Luciferase cLuc; red:Myc tag; dark blue: gs linker; black: N7
nLuc:myc:gs linker:N8
MGSGEDAKNIKKGPAPFYPLEDGTAGEQLHKAMKRYALVPGTIAFTDAHIEVDITYAEYFEMSVRLAEAMKRYGLNTNHRIVVCSENSLQFFMPVLGALFIGVAVAPAND<br>IYNERELLNSMGISQPTVVFVSKKGLQKILNVQKKLPIIQKIIIMDSKTDYQGFQSMYTFVTSHLPPGFNEYDFVPESFDRDKTIALIMNSSGSTGLPKGVALPHRTACV<br>RFSHARDPIFGNQIIPDTAILSVVPFHHGFGMFTTLGYLICGFRVVLMYRFEEELFLRSLQDYKIQSALLVPTLFSFFAKSTLIDKYDLSNLHEIASGGAPLSKEVGEAV<br>AKRFHLPGIRQGYGLTETTSAILITPEGDDKPGAVGKVVPFFEAKVVDLDTGKTLGVNQRGELCVRGPMIMSGYVNNPEATNALIDKDGWLHSGDIAYWDEDEHFFIVDR<br>LKSLIKYKGYQVAPAELESILLQHPNIFDAGVAGLPDDDAGELPAAVVVLEHGK EQKLISEEDL GGSGG YGKIAALKAENAALEAKIAALKAEIAALEAGY*
purple: N terminal of split luciferase nLuc; red: Myc tag; dark blue: gs linker; black: N8
N8:myc: gs linker:nLuc
M YGKIAALKAENAALEAKIAALKAEIAALEAGY EQKLISEEDL GGSGGGSG<br>EDAKNIKKGPAPFYPLEDGTAGEQLHKAMKRYALVPGTIAFTDAHIEVDITYAEYFEMSVRLAEAMKRYGLNTNHRIVVCSENSLQFFMPVLGALFIGVAVAPANDIYNE<br>RELLNSMGISQPTVVFVSKKGLQKILNVQKKLPIIQKIIIMDSKTDYQGFQSMYTFVTSHLPPGFNEYDFVPESFDRDKTIALIMNSSGSTGLPKGVALPHRTACVRFSH<br>ARDPIFGNQIIPDTAILSVVPFHHGFGMFTTLGYLICGFRVVLMYRFEEELFLRSLQDYKIQSALLVPTLFSFFAKSTLIDKYDLSNLHEIASGGAPLSKEVGEAVAKRF<br>HLPGIRQGYGLTETTSAILITPEGDDKPGAVGKVVPFFEAKVVDLDTGKTLGVNQRGELCVRGPMIMSGYVNNPEATNALIDKDGWLHSGDIAYWDEDEHFFIVDRLKSL<br>IKYKGYQVAPAELESILLQHPNIFDAGVAGLPDDDAGELPAAVVVLEHGK*
purple: N terminal of split luciferase nLuc; red: Myc tag; dark blue: gs linker; black: N8
His:TALE-A:NLS:gs lineker:N8
M HHHHHH<br>DYKDHDGDYKDHDIDYKDDDDKMAPKKKRKVGIHRGVPMVDLRTLGYSQQQQEKIKPKVRSTVAQHHEALVGHGFTHAHIVALSQHPAALGTVAVKYQDMIAALPEATHE<br>AIVGVGKQWSGARALEALLTVAGELRGPPLQLDTGQLLKIAKRGGVTAVEAVHAWRNALTGAPLNLTPDQVVAIASNGGGKQALETVQRLLPVLCQDHGLTPEQVVAIAS<br>NGGGKQALETVQRLLPVLCQAHGLTPDQVVAIASNIGGKQALETVQRLLPVLCQAHGLTPAQVVAIASHDGGKQALETVQRLLPVLCQDHGLTPDQVVAIASNGGGKQAL

ETVQRLLPVLCQDHGLTPEQVVAIANNNGGKQALETVQRLLPVLCQAHGLTPDQVVAIASHDGGKQALETVQRLLPVLCQAHGLTPAQVVAIASNGGGKQALETVQRLLP
VLCQDHGLTPDQVVAIANNNGGKQALETVQRLLPVLCQDHGLTPEQVVAIASHDGGKQALETVQRLLPVLCQAHGLTPDQVVAIASNGGGKQALETVQRLLPVLCQAHGL
TPAQVVAIASHDGGKQALETVQRLLPVLCQDHGLTPDQVVAIASHDGGKQALETVQRLLPVLCQDHGLTPEQVVAIASHDGGKQALETVQRLLPVLCQAHGLTPDQVVAI
ANNNGGKQALETVQRLLPVLCQAHGLTPAQVVAIASHDGGKQALETVQRLLPVLCQDHGLTPEQVVAIASNGGGRPALESIVAQLSRPDPALAALTNDHLVALACLGGRP
ALDAVKKGLPHAPALIKRTNRRIPERTSHRVAGS DPKKKRKV GGSGGGSGGS YKIAALKAENAALEAKIAALKAEIAALEAGC*

magenta: his tag; orange: TALA; dark blue: gs linker; cian: nuclear localization sequence; black: N8

N7:gs linker:NLS:VP16:[AU1]

M GEIAALEAKNAALKAEIAALEAKIAALKAGY GGSGGGSGGS DPKKKRKV
APPTDVSLGDELHLDGEDVAMAHADALDDFDLDMLGDGDSPGPGFTPHDSAPYGALDMADFEFEQMFTDALGIDEYGG DTYRYI*

black:N7; dark blue:gs linker; cian: nuclear localization sequence; dark green VP16 activation domain; light green: AU1 tag

N7Q:gs linker:NLS:VP16:[AU1]

M GEIAALEQKNAALKQEIAALEQKIAALKQGC GGSGGGSGGS DPKKKRKV
APPTDVSLGDELHLDGEDVAMAHADALDDFDLDMLGDGDSPGPGFTPHDSAPYGALDMADFEFEQMFTDALGIDEYGG DTYRYI*

black:N7Q; dark blue:gs linker; cian: nuclear localization sequence; dark green VP16 activation domain; light green: AU1 tag

P7A:gs linker:NLS:VP16:[AU1]

M GEIAALEAKNAALKAEIAALEAKNAALKAGC GGSGGGSGGS DPKKKRKV
APPTDVSLGDELHLDGEDVAMAHADALDDFDLDMLGDGDSPGPGFTPHDSAPYGALDMADFEFEQMFTDALGIDEYGG DTYRYI*

black:P7A; dark blue:gs linker; cian: nuclear localization sequence; dark green VP16 activation domain; light green: AU1 tag

P7:gs linker:NLS:VP16:[AU1]

M EIQALEEKNAQLKQEIAALEEKNQALKYG GGSGGGSGGS DPKKKRKV
APPTDVSLGDELHLDGEDVAMAHADALDDFDLDMLGDGDSPGPGFTPHDSAPYGALDMADFEFEQMFTDALGIDEYGG DTYRYI*

black:P7; dark blue:gs linker; cian: nuclear localization sequence; dark green VP16 activation domain; light green: AU1 tag

P7SN:gs linker:NLS:VP16:[AU1]

M EIQQLEEKNSQLKQEISQLEEKNQELKY GGSGGGSGGS DPKKKRKV
APPTDVSLGDELHLDGEDVAMAHADALDDFDLDMLGDGDSPGPGFTPHDSAPYGALDMADFEFEQMFTDALGIDEYGG DTYRYI*

black:P7SN; dark blue:gs linker; cian: nuclear localization sequence; dark green VP16 activation domain; light green: AU1 tag

Myc:N7:NLS

M EQKLISEEDL GEIAALEAKNAALKAEIAALEAKIAALKAGY DPKKKRKV*

red: Myc tag; Black: N7; cian: nuclear localization sequence

Myc:N7Q:NLS

M EQKLISEEDL GEIAALEQKNAALKQEIAALEQKIAALKQGC DPKKKRKV*

red: Myc tag; Black: N7Q; cian: nuclear localization sequence

Myc:P7A:NLS

M EQKLISEEDL GEIAALEAKNAALKAEIAALEAKNAALKAGC DPKKKRKV*

red: Myc tag; Black: P7A; cian: nuclear localization sequence

Myc:P7:NLS

M EQKLISEEDL EIQALEEKNAQLKQEIAALEEKNQALKYG DPKKKRKV*

red: Myc tag; Black: P7; cian: nuclear localization sequence

Myc:P7SN:NLS

M EQKLISEEDL EIQQLEEKNSQLKQEISQLEEKNQELKYG DPKKKRKV*

red: Myc tag; Black: P7SN; cian: nuclear localization sequence

nLuc:P8A

MGSGEDAKNIKKGPAPFYPLEDGTAGEQLHKAMKRYALVPGTIAFTDAHIEVDITYAEYFEMSVRLAEAMKRYGLNTNHRIVVCSENSLQFFMPVLGALFIGVAVAPAND
IYNERELLNSMGISQPTVVFVSKKGLQKILNVQKKLPIIQKIIIMDSKTDYQGFQSMYTFVTSHLPPGFNEYDFVPESFDRDKTIALIMNSSGSTGLPKGVALPHRTACV
RFSHARDPIFGNQIIPDTAILSVVPFHHGFGMFTTLGYLICGFRVVLMYRFEEELFLRSLQDYKIQSALLVPTLFSFFAKSTLIDKYDLSNLHEIASGGAPLSKEVGEAV
AKRFHLPGIRQGYGLTETTSAILITPEGDDKPGAVGKVVPFFEAKVVDLDTGKTLGVNQRGELCVRGPMIMSGYVNNPEATNALIDKDGWLHSGDIAYWDEDEHFFIVDR
LKSLIKYKGYQVAPAELESILLQHPNIFDAGVAGLPDDDAGELPAAVVVLEHGK EQKLISEEDL GGSGG KIAALKAENAALEAKIAALKAENAALEAGGC

purple: N terminal of split luciferase nLuc; red: Myc tag; dark blue: gs linker; black: P8A

cLuc:N5

M TMTEKEIVDYVASQVTTAKKLRGGVVFVDEVPKGLTGKLDARKIREILIKAKKGGKIAVN EQKLISEEDL GEIAALEAKIAALKAKNAALKAEIAALEAGY

yellow: C terminal of split Luciferase cLuc; red:Myc tag; dark blue: gs linker; black: N5

cLuc:P5A

M TMTEKEIVDYVASQVTTAKKLRGGVVFVDEVPKGLTGKLDARKIREILIKAKKGGKIAVN EQKLISEEDL ENAALEAKIAALKAKNAALKAEIAALEA

yellow: C terminal of split Luciferase cLuc; red:Myc tag; dark blue: gs linker; black: P5A

cLuc:P5

M TMTEKEIVDYVASQVTTAKKLRGGVVFVDEVPKGLTGKLDARKIREILIKAKKGGKIAVN EQKLISEEDL SPEDENAALEEKIAQLKQKNAALKEEIQALEYG

yellow: C terminal of split Luciferase cLuc; red:Myc tag; dark blue: gs linker; black: P5

cLuc:myc:P5SN

M TMTEKEIVDYVASQVTTAKKLRGGVVFVDEVPKGLTGKLDARKIREILIKAKKGGKIAVN EQKLISEEDL ENSQLEEKISQLKQKNSELKEEIQQLE

yellow: C terminal of split Luciferase cLuc; red:Myc tag; dark blue: gs linker; black: P5SN

nLuc:P6A

MGSGEDAKNIKKGPAPFYPLEDGTAGEQLHKAMKRYALVPGTIAFTDAHIEVDITYAEYFEMSVRLAEAMKRYGLNTNHRIVVCSENSLQFFMPVLGALFIGVAVAPAND
IYNERELLNSMGISQPTVVFVSKKGLQKILNVQKKLPIIQKIIIMDSKTDYQGFQSMYTFVTSHLPPGFNEYDFVPESFDRDKTIALIMNSSGSTGLPKGVALPHRTACV
RFSHARDPIFGNQIIPDTAILSVVPFHHGFGMFTTLGYLICGFRVVLMYRFEEELFLRSLQDYKIQSALLVPTLFSFFAKSTLIDKYDLSNLHEIASGGAPLSKEVGEAV
AKRFHLPGIRQGYGLTETTSAILITPEGDDKPGAVGKVVPFFEAKVVDLDTGKTLGVNQRGELCVRGPMIMSGYVNNPEATNALIDKDGWLHSGDIAYWDEDEHFFIVDR
LKSLIKYKGYQVAPAELESILLQHPNIFDAGVAGLPDDDAGELPAAVVVLEHGK EQKLISEEDL GGSGG GKNAALKAEIAALEAENAALEAKIAALKAGGC

purple: N terminal of split luciferase nLuc; red: Myc tag; dark blue: gs linker; black:P6A

nLuc:N6

MGSGEDAKNIKKGPAPFYPLEDGTAGEQLHKAMKRYALVPGTIAFTDAHIEVDITYAEYFEMSVRLAEAMKRYGLNTNHRIVVCSENSLQFFMPVLGALFIGVAVAPAND
IYNERELLNSMGISQPTVVFVSKKGLQKILNVQKKLPIIQKIIIMDSKTDYQGFQSMYTFVTSHLPPGFNEYDFVPESFDRDKTIALIMNSSGSTGLPKGVALPHRTACV
RFSHARDPIFGNQIIPDTAILSVVPFHHGFGMFTTLGYLICGFRVVLMYRFEEELFLRSLQDYKIQSALLVPTLFSFFAKSTLIDKYDLSNLHEIASGGAPLSKEVGEAV
AKRFHLPGIRQGYGLTETTSAILITPEGDDKPGAVGKVVPFFEAKVVDLDTGKTLGVNQRGELCVRGPMIMSGYVNNPEATNALIDKDGWLHSGDIAYWDEDEHFFIVDR
LKSLIKYKGYQVAPAELESILLQHPNIFDAGVAGLPDDDAGELPAAVVVLEHGK EQKLISEEDL GGSGG KIAALKAEIAALEAENAALEAKIAALKAG*

purple: N terminal of split luciferase nLuc; red: Myc tag; dark blue: gs linker; black:N6

N5:gs linker:NLS:VP16:[AU1]

M GEIAALEAKIAALKAKNAALKAEIAALEAGY GGSGGGSGGS DPKKKRKV
APPTDVSLGDELHLDGEDVAMAHADALDDFDLDMLGDGDSPGPGFTPHDSAPYGALDMADFEFEQMFTDALGIDEYGG DTYRYI*

black:N5; dark blue:gs linker; cian: nuclear localization sequence; dark green VP16 activation domain; light green: AU1 tag

P5A: gs linker:NLS:VP16:^{AU1}

M ENAALEAKIAALKAKNAALKAEIAALEA GGSGGGSGGS DPKKKRKV
APPTDVSLGDELHLDGEDVAMAHADALDDFDLDMLGDGDSPGPGFTPHDSAPYGALDMADFEFEQMFTDALGIDEYGG DTYRYI*

black:P5A; dark blue:gs linker; cian: nuclear localization sequence; dark green VP16 activation domain; light green: AU1 tag

P5: gs linker:NLS:VP16:^{AU1}

M SPEDENAALEEKIAQLKQKNAALKEEIQALEYG GGSGGGSGGS DPKKKRKV
APPTDVSLGDELHLDGEDVAMAHADALDDFDLDMLGDGDSPGPGFTPHDSAPYGALDMADFEFEQMFTDALGIDEYGG DTYRYI*

black:P5; dark blue:gs linker; cian: nuclear localization sequence; dark green VP16 activation domain; light green: AU1 tag

P5SN: gs linker:NLS:VP16:^{AU1}

M ENSQLEEKISQLKQKNSELKEEIQQLE GGSGGGSGGS DPKKKRKV
APPTDVSLGDELHLDGEDVAMAHADALDDFDLDMLGDGDSPGPGFTPHDSAPYGALDMADFEFEQMFTDALGIDEYGG DTYRYI*

black:CC; dark blue:gs linker; cian: nuclear localization sequence; dark green VP16 activation domain; light green: AU1 tag

Myc:N5^{NLS}

M EQKLISEEDL GEIAALEAKIAALKAKNAALKAEIAALEAGY DPKKKRKV*

red: Myc tag; Black: N5; cian: nuclear localization sequence

Myc:P5A^{NLS}

M EQKLISEEDL ENAALEAKIAALKAKNAALKAEIAALEA DPKKKRKV*

red: Myc tag; Black: P5A; cian: nuclear localization sequence

Myc:P5^{NLS}

M EQKLISEEDL SPEDENAALEEKIAQLKQKNAALKEEIQALEYG DPKKKRKV*

red: Myc tag; Black: P5; cian: nuclear localization sequence

Myc:P5SN^{NLS}

M EQKLISEEDL ENSQLEEKISQLKQKNSELKEEIQQLE DPKKKRKV*

red: Myc tag; Black: P5SN; cian: nuclear localization sequence

Myc:N5:KRAB

M EQKLISEEDL GEIAALEAKIAALKAKNAALKAEIAALEAGY DPKKKRKV
PKKKRKVDGGGALSPQHSAVTQGSIIKNKEGMDAKSLTAWSRTLVTFKDVFVDFTREEWKLLDTAQQIVYRNVMLENYKNLVSLGYQLTKPDVILRLEKGEEPWLVEREI
HQETHPDSETAFEIKSSV DTYRYI*

red: Myc tag; Black: N5; cian: nuclear localization sequence; orange: KRAB suppressino domain; light green: AU1 tag

Myc:P5A:KRAB

M EQKLISEEDL ENAALEAKIAALKAKNAALKAEIAALEA DPKKKRKV
PKKKRKVDGGGALSPQHSAVTQGSIIKNKEGMDAKSLTAWSRTLVTFKDVFVDFTREEWKLLDTAQQIVYRNVMLENYKNLVSLGYQLTKPDVILRLEKGEEPWLVEREI
HQETHPDSETAFEIKSSV DTYRYI*

red: Myc tag; Black: P5A; cian: nuclear localization sequence; orange: KRAB suppressino domain; light green: AU1 tag

Myc:P5:KRAB

M EQKLISEEDL SPEDENAALEEKIAQLKQKNAALKEEIQALEYG DPKKKRKV
PKKKRKVDGGGALSPQHSAVTQGSIIKNKEGMDAKSLTAWSRTLVTFKDVFVDFTREEWKLLDTAQQIVYRNVMLENYKNLVSLGYQLTKPDVILRLEKGEEPWLVEREI
HQETHPDSETAFEIKSSV DTYRYI*

red: Myc tag; Black: P5; cian: nuclear localization sequence; orange: KRAB suppressino domain; light green: AU1 tag

Myc:P5SN:KRAB

M EQKLISEEDL ENSQLEEKISQLKQKNSELKEEIQQLE DPKKKRKV
PKKKRKVDGGGALSPQHSAVTQGSIIKNKEGMDAKSLTAWSRTLVTFKDVFVDFTREEWKLLDTAQQIVYRNVMLENYKNLVSLGYQLTKPDVILRLEKGEEPWLVEREI
HQETHPDSETAFEIKSSV DTYRYI*

red: Myc tag; Black: P5SN; cian: nuclear localization sequence; orange: KRAB suppressino domain; light green: AU1 tag

His:TALE-A:NLS:gs lineker:N6 (TALE-A:N6)

M HHHHHH
DYKDHDGDYKDHDIDYKDDDDKMAPKKKRKVGIHRGVPMVDLRTLGYSQQQQEKIKPKVRSTVAQHHEALVGHGFTHAHIVALSQHPAALGTVAVKYQDMIAALPEATHE
AIVGVGKQWSGARALEALLTVAGELRGPPLQLDTGQLLKIAKRGGVTAVEAVHAWRNALTGAPLNLTPDQVVAIASNGGGKQALETVQRLLPVLCQDHGLTPEQVVAIAS
NGGGKQALETVQRLLPVLCQAHGLTPDQVVAIASNIGGKQALETVQRLLPVLCQAHGLTPAQVVAIASHDGGKQALETVQRLLPVLCQDHGLTPDQVVAIASNGGGKQAL
ETVQRLLPVLCQDHGLTPEQVVAIANNNGGKQALETVQRLLPVLCQAHGLTPDQVVAIASHDGGKQALETVQRLLPVLCQAHGLTPAQVVAIASNGGGKQALETVQRLLP
VLCQDHGLTPDQVVAIANNNGGKQALETVQRLLPVLCQDHGLTPEQVVAIASHDGGKQALETVQRLLPVLCQAHGLTPDQVVAIASNGGGKQALETVQRLLPVLCQAHGL
TPAQVVAIASHDGGKQALETVQRLLPVLCQDHGLTPDQVVAIASHDGGKQALETVQRLLPVLCQDHGLTPEQVVAIASHDGGKQALETVQRLLPVLCQAHGLTPDQVVAI
ANNNGGKQALETVQRLLPVLCQAHGLTPAQVVAIASHDGGKQALETVQRLLPVLCQDHGLTPEQVVAIASNGGGRPALESIVAQLSRPDPALAALTNDHLVALACLGGRP
ALDAVKKGLPHAPALIKRTNRRIPERTSHRVAGS DPKKKRKV GGSGGGSGGS KIAALKAEIAALEAENAALEAKIAALKAG*

magenta: his tag; orange: TALA; dark blue: gs linker; cian: nuclear localization sequence; black: N6

His:TALE-A:NLS:gs lineker:N6:linker:N8 (TALE-A:N6:N8)

M HHHHHH
DYKDHDGDYKDHDIDYKDDDDKMAPKKKRKVGIHRGVPMVDLRTLGYSQQQQEKIKPKVRSTVAQHHEALVGHGFTHAHIVALSQHPAALGTVAVKYQDMIAALPEATHE
AIVGVGKQWSGARALEALLTVAGELRGPPLQLDTGQLLKIAKRGGVTAVEAVHAWRNALTGAPLNLTPDQVVAIASNGGGKQALETVQRLLPVLCQDHGLTPEQVVAIAS
NGGGKQALETVQRLLPVLCQAHGLTPDQVVAIASNIGGKQALETVQRLLPVLCQAHGLTPAQVVAIASHDGGKQALETVQRLLPVLCQDHGLTPDQVVAIASNGGGKQAL
ETVQRLLPVLCQDHGLTPEQVVAIANNNGGKQALETVQRLLPVLCQAHGLTPDQVVAIASHDGGKQALETVQRLLPVLCQAHGLTPAQVVAIASNGGGKQALETVQRLLP
VLCQDHGLTPDQVVAIANNNGGKQALETVQRLLPVLCQDHGLTPEQVVAIASHDGGKQALETVQRLLPVLCQAHGLTPDQVVAIASNGGGKQALETVQRLLPVLCQAHGL
TPAQVVAIASHDGGKQALETVQRLLPVLCQDHGLTPDQVVAIASHDGGKQALETVQRLLPVLCQDHGLTPEQVVAIASHDGGKQALETVQRLLPVLCQAHGLTPDQVVAI
ANNNGGKQALETVQRLLPVLCQAHGLTPAQVVAIASHDGGKQALETVQRLLPVLCQDHGLTPEQVVAIASNGGGRPALESIVAQLSRPDPALAALTNDHLVALACLGGRP
ALDAVKKGLPHAPALIKRTNRRIPERTSHRVAGS DPKKKRKV GGSGGGSGGS KIAALKAEIAALEAENAALEAKIAALKAG GGSGGGSGGS
YKIAALKAENAALEAKIAALKAEIAALEAGC*

magenta: his tag; orange: TALA; dark blue: gs linker; cian: nuclear localization sequence; black: N6 and N8

CoV-2 Spike-protein

M
FLLTTKRTMFVFLVLLPLVSSQCVNLTTRTQLPPAYTNSFTRGVYYPDKVFRSSVLHSTQDLFLPFFSNVTWFHAIHVSGTNGTKRFDNPVLPFNDGVYFASTEKSNIIR
GWIFGTTLDSKTQSLLIVNNATNVVIKVCEFQFCNDPFLGVYYHKNNKSWMESEFRVYSSANNCTFEYVSQPFLMDLEGKQGNFKNLREVFKNIDGYFKIYSKHTPINLV
RDLPQGFSALEPLVDLPIGINITRFQTLLALHRSYLTPGDSSSGWTAGAAAYYVGYLQPRTFLLKYNENGTITDAVDCALDPLSETKCTLKSFTVEKGIYQTSNFRVQPT
ESIVRFPNITNLCPFGEVFNATRFASVYAWNRKRISNCVADYSVLYNSASFSTFKCYGVSPTKLNDLCFTNVYADSFVIRGDEVRQIAPGQTGKIADYNYKLPDDFTGCV
IAWNSNNLDSKVGGNYNYLYRLFRKSNLKPFERDISTEIYQAGSTPCNGVEGFNCYFPLQSYGFQPTNGVGYQPYRVVVLSFELLHAPATVCGPKKSTNLVKNKCVNFNF

NGLTGTGVLTESNKKFLPFQQFGRDIADTTDAVRDPQTLEILDITPCSFGGVSVITPGTNTSNQVAVLYQDVNCTEVPVAIHADQLTPTWRVYSTGSNVFQTRAGCLIGA
EHVNNSYECDIPIGA
GICASYQTQTNSPRRARSVASQSIIAYTMSLGAENSVAYSNNSIAIPTNFTISVTTEILPVSMTKTSVDCTMYICGDSTECSNLLLQYGSFCTQLNRALTGIAVEQDKNT
QEVFAQVKQIYKTPPIKDFGGFNFSQILPDPSKPSKRSFIEDLLFNKVTLADAGFIKQYGDCLGDIAARDLICAQKFNGLTVLPPLLTDEMIAQYTSALLAGTITSGWTF
GAGAALQIPFAMQMAYRFNGIGVTQNVLYENQKLIANQFNSAIGKIQDSLSSTASALGKLQDVVNQNAQALNTLVKQLSSNFGAISSVLNDILSRLDKVEAEVQIDRLIT
GRLQSLQTYVTQQLIRAAEIRASANLAATKMSECVLGQSKRVDFCGKGYHLMSFPQSAPHGVVFLHVTYVPAQEKNFTTAPAICHDGKAHFPREGVFVSNGTHWFVTQRN
FYEPQIITTDNTFVSGNCDVVIGIVNNTVYDPLQPELDSFKEELDKYFKNHTSPDVDLGDISGINASVVNIQKEIDRLNEVAKNLNESLIDLQELGKYEQYIKWPWYIWL
GFIAGLIAIVMVTIMLCCMTSCCSCLKGCCSCGSCCKFDEDDSEPVLKGVKLHYT*

Dark red:S1; blue: S2

hACE2

MSSSSWLLLSLVAVTAAQSTIEEQAKTFLDKFNHEAEDLFYQSSLASWNYNTNITEENVQNMNNAGDKWSAFLKEQSTLAQMYPLQEIQNLTVKLQLQALQQNGSSVLSE
DKSKRLNTILNTMSTIYSTGKVCNPDNPQECLLLEPGLNEIMANSLDYNERLWAWESWRSEVGKQLRPLYEEYVVLKNEMARANHYEDYGDYWRGDYEVNGVDGYDYSRG
QLIEDVEHTFEEIKPLYEHLHAYVRAKLMNAYPSYISPIGCLPAHLLGDMWGRFWTNLYSLTVPFGQKPNIDVTDAMVDQAWDAQRIFKEAEKFFVSVGLPNMTQGFWEN
SMLTDPGNVQKAVCHPTAWDLGKGDFRILMCTKVTMDDFLTAHHEMGHIQYDMAYAAQPFLLRNGANEGFHEAVGEIMSLSAATPKHLKSIGLLSPDFQEDNETEINFLL
KQALTIVGTLPFTYMLEKWRWMVFKGEIPKDQWMKKWWEMKREIVGVVEPVPHDETYCDPASLFHVSNDYSFIRYYTRTLYQFQFQEALCQAAKHEGPLHKCDISNSTEA
GQKLFNMLRLGKSEPWTLALENVVGAKNMNVRPLLNYFEPLFTWLKDQNKNSFVGWSTDWSPYADQSIKVRISLKSALGDKAYEWNDNEMYLFRSSVAYAMRQYFLKVKN
QMILFGEEDVRVANLKPRISFNFFVTAPKNVSDIIPRTEVEKAIRMSRSRINDAFRLNDNSLEFLGIQPTLGPPNQPPVSIWLIVFGVVMGVIVVGIVILIFTGIRDRKK
KNKARSGENPYASIDISKGENNPGFQNTDDVQTSF*

hTMPRSS2 (SinoBiological)

MALNSGSPPAIGPYYENHGYQPENPYPAQPTVVPTVYEVHPAQYYPSPVPQYAPRVLTQASNPVVCTQPKSPSGTVCTSKTKKALCITLTLGTFLVGAALAAGLLWKFMG
SKCSNSGIECDSSGTCINPSNWCDGVSHCPGGEDENRCVRLYGPNFILQVYSSQRKSWHPVCQDDWNENYGRAACRDMGYKNNFYSSQGIVDDSGSTSFMKLNTSAGNVD
IYKKLYHSDACSSKAVVSLRCIACGVNLNSSRQSRIVGGESALPGAWPWQVSLHVQNVHVCGGSIITPEWIVTAAHCVEKPLNNPWHWTAFAGILRQSFMFYGAGYQVEK
VISHPNYDSKTKNNDIALMKLQKPLTFNDLVKPVCLPNPGMMLQPEQLCWISGWGATEEKGKTSEVLNAAKVLLIETQRCNSRYVYDNLITPAMICAGFLQGNVDSCQGD
SGGPLVTSKNNIWWLIGDTSWGSGCAKAYRPGVYGNVMVFTDWIYRQMRADG GGGS EQKLISEEDL*

Black: hMPRSS2; dark blue: gs linker; red:Myc tag

GFP$_{1-10}$:gs linker:N7 (GFP$_{1-10}$:N7)

SKGEELFTGVVPILVELDGDVNGHKFSVRGEGEGDATIGKLTLKFICTTGKLPVPWPTLVTTLTYGVQCFSRYPDHMKRHDFFKSAMPEGYVQERTISFKDDGKYKTRAV
VKFEGDTLVNRIELKGTDFKEDGNILGHKLEYNFNSHNVYITADKQKNGIKANFTVRHNVEDGSVQLADHYQQNTPIGDGPVLLPDNHYLSTQTVLSKDPNEKSGSGSGS
GSSGGSGSGEIAALEAKNAALKAEIAALEAKIAALKAGY*

Green: GFP(1-10); dark blue: gs Linker; black:N7

GFP$_{1-10}$:gs linker:P7 (GFP$_{1-10}$:P7)

SKGEELFTGVVPILVELDGDVNGHKFSVRGEGEGDATIGKLTLKFICTTGKLPVPWPTLVTTLTYGVQCFSRYPDHMKRHDFFKSAMPEGYVQERTISFKDDGKYKTRAV
VKFEGDTLVNRIELKGTDFKEDGNILGHKLEYNFNSHNVYITADKQKNGIKANFTVRHNVEDGSVQLADHYQQNTPIGDGPVLLPDNHYLSTQTVLSKDPNEKSGSGSGS
GSSGGSGSGEIQALEEKNAQLKQEIAALEEKNQALKYG*

Green: GFP(1-10); dark blue: gs Linker; black:P7

^{His}N8:gs linker:GFP$_{11}$ (N8:GFP$_{11}$)

M HHHHHH YGKIAALKAENAALEAKIAALKAEIAALEAGY GGSGGGSG RDHMVLHEYVNAAGIT

magenta: his tag; dark blue: gs linker; black: N8; green: GFP11;

^{His}P8:gs linker:GFP$_{11}$ (P8:GFP$_{11}$)

M HHHHHH KIAQLKE ENQQLEQ KIQALKE ENAALEY GGSGGGSG RDHMVLHEYVNAAGIT

magenta: his tag; dark blue: gs linker; black: P8; green: GFP11

^{His}(N8:gs linker:GFP$_{11}$)$_3$ (3×(N8:GFP$_{11}$))

M HHHHHH YGKIAALKAENAALEAKIAALKAEIAALEAGY GGSGGGSG RDHMVLHEYVNAAGIT GSGGGSG YGKIAALKAENAALEAKIAALKA EIAALEAGY
GGSGGGSG RDHMVLHEYVNAAGIT GGSGGGSG YGKIAALKAENAALEAKIAALKAEIAALEAGY GGSGGGSG RDHMVLHEYVNAAGIT

magenta: his tag; dark blue: gs linker; black: N8; green: GFP11
